# *Aspergillus terreus* Inhibits Growth and Induces Morphological Abnormalities in *Pythium aphanidermatum* and Suppresses *Pythium*-Induced Damping-Off of Cucumber

**DOI:** 10.3389/fmicb.2018.00095

**Published:** 2018-02-01

**Authors:** Boshra A. Halo, Rashid A. Al-Yahyai, Abdullah M. Al-Sadi

**Affiliations:** Department of Crop Sciences, College of Agricultural and Marine Sciences, Sultan Qaboos University, Muscat, Oman

**Keywords:** biocontrol, antagonistic activity, cucumber damping-off, oomycete, biological control

## Abstract

The study investigated the efficacy of two isolates of *Aspergillus terreus* (65P and 9F) on the growth, morphology and pathogenicity of *Pythium aphanidermatum* on cucumber. *In vitro* tests showed that the two isolates inhibited the growth of *P. aphanidermatum* in culture. Investigating *P. aphanidermatum* hyphae close to the inhibition zone showed that the hyphae showed abnormal growth and loss of internal content. Treating *P. aphanidermatum* with the culture filtrate (CF) of *A. terreus* resulted in significant rise in cellular leakage of *P. aphanidermatum* mycelium. Testing glucanase enzyme activity by both *A. terreus* isolates showed a significant increase in glucanase activity. This suggests that the cell walls of *Pythium*, which consist of glucan, are affected by the glucanase enzyme produced by *A. terreus*. In addition, Aspergillus isolates produced siderephore, which is suggested to be involved in inhibition of *Pythium* growth. Also, the CFs of 65P and 9F isolates significantly reduced spore production by *P. aphanidermatum* compared to the control (*P* < 0.05). In bioassay tests, the two isolates of *A. terreus* increased the survival rate of cucumber seedlings from 10 to 20% in the control seedlings treated with *P. aphanidermatum* to 38–39% when the biocontrol agents were used. No disease symptoms were observed on cucumber seedlings only treated with the isolates 65P and 9F of *A. terreus*. In addition, the *A. terreus* isolates did not have any negative effects on the growth of cucumber seedlings. This study shows that isolates of *A. terreus* can help suppress *Pythium*-induced damping-off of cucumber, which is suggested to be through the effect of *A. terreus* and its glucanase enzyme on *P. aphanidermatum* mycelium.

## Introduction

In Oman, over 90% of greenhouses are dedicated exclusively for cucumber (*Cucumis sativus*). However, cucumber production suffers from *Pythium*-induced damping-off disease which is responsible for over 75% mortality in cucumber seedlings (Al-Kiyumi, 2006; Al-Sadi et al., 2012). Damping-off of vegetable crops is caused by several species of *Pythium* (Kraus and Loper, 1992; Al-Sadi et al., 2010), *Rhizoctonia solani* (Asaka and Shoda, 1996; Sadeghi et al., 2006), *Phytophthora capsici* (Sharifi-Tehrani and Omati, 1999), and *Fusarium* (Berg et al., 2017; Hrunyk et al., 2017; Lamprecht and Tewoldemedhin, 2017). *P. aphanidermatum* is the main causal agent of damping-off of cucumber in Oman and elsewhere (Al-Sadi et al., 2011, 2012; Hatami et al., 2013).

Several methods are used to manage damping-off disease, which include chemical, physical and biological methods. Chemical control of Pythium damping-off is practiced through the use of Mefenoxam, Hymexazol, Propamocarb, and other fungicides (Papavizas et al., 1978; Al-Sa’di et al., 2008, Al-Sadi et al., 2015a). Growers usually use solarization in summer to reduce Pythium propagules in soil (Deadman et al., 2007). Biological control, using microorganisms to inhibit plant pathogens, offers another alternative to chemical control. Several studies indicated the successful use of biocontrol agents to suppress *Pythium* damping-off of cucumber. These include the use of biocontrol agents such as *Pseudomonas fluorescens*, *Trichoderma harzianum*, and *Penicillium stipitatum* (Georgakopoulos et al., 2002; Al-Hinai et al., 2010; Al-Sadi et al., 2015b).

Endophytic fungi promote biotic stress tolerance, including disease stress, to host plants and they have critical roles in plant survival under stress conditions. Various endophytic fungi and bacteria, including *Actinomycetes, Bacillus*, *Pseudomonas*, *Trichoderma*, and *Epicoccum* were reported to elicit plant disease tolerance in tomato, cotton, chilli, potato and cacao (Rajendran and Samiyappan, 2008; Lahlali and Hijri, 2010; Muthukumar et al., 2010; Goudjal et al., 2014). There are several mechanisms followed by endophytic fungi and bacteria in the biocontrol of pathogens. These include the synthesis of secondary metabolites such as antibacterial, antifungal and anti-insect substances (Xiao et al., 2014; Mousa et al., 2016; Burgess et al., 2017), competition in rhizosphere (Weller, 1988; Whipps, 2001) and the induction of defense responses in plants against pathogens (Yedidia et al., 1999; Howell, 2003). Others play an important role in mineral and element solubilisation for plant absorption and nutrition (Wakelin et al., 2004; Zhang et al., 2013).

*Aspergillus terreus* is a common fungus in soil and plants (Tarafdar et al., 1988; Khan et al., 2010). A study showed that an antifungal compound from *A. terreus* effectively inhibited the phytopathogenic fungi *Botrytis cinerea*, *Rhizoctonia solani*, and *Pythium ultimum* (Kim et al., 1998). Another study revealed the effect of *Aspergillus* species bioactive metabolites on *Pythium ultimum* control (Abdallah et al., 2014). Moreover, applying *A. terreus* provided effective disease control to soil infested with *P. deliense* that causes damping-off disease of maize (Abdelzaher et al., 2000). Also, the combined treatment of *A. terreus* and *Acremonium strictum* led to antagonistic influence on root-knot disease of tomato caused by *Meloidogyne incognita* (Singh and Mathur, 2010).

To our knowledge, the efficacy of *A. terreus* to control *P. aphanidermatum*-induced damping-off of cucumber has not yet been reported, therefore, this study aims to investigate the ability of the endophytic fungus *A. terreus* to suppress *P. aphanidermatum* and *Pythium*-induced damping-off of cucumber. Objectives of this research work were (1) to select and identify endophytic fungi from plants in Oman which are effective in suppressing *P. aphanidermatum*; (2) to determine the morphological changes of *P. aphanidermatum* under *A. terreus* treatments using light microscope and scanning electron microscope; (3) to determine the effect of *A. terreus* culture filtrate (CF) on spore production and cellular leakage of *P. aphanidermatum*; and (4) to investigate the ability of *A. terreus* in the biocontrol of damping-off of cucumber. The selection of *Rhazya stricta* and *Tephrosia apollinea* plants for the isolation of endophytic fungi was mainly due to the intention to isolate endophytes which are present in/on native plants, and not on cultivated plants on which endophytes might be introduced from abroad. This will help come up with antagonistic fungi adapted to conditions of this part of the world.

## Materials and Methods

### Collection, Isolation, and Identification of *Aspergillus* Isolates

Fresh plants of *Rhazya stricta* and *Tephrosia apollinea* were collected from Haima and Adam, in the Sultanate of Oman in May 2016. Endophytic fungi present in the samples were isolated using a modified method of Larran et al. (2002). Briefly, root, shoot and leaves were washed under running tap water and cut into several pieces (approximately 5 mm diameter). Then, they were surface sterilized by dipping successively into 70% ethanol for 1 min, sodium hypochlorite 1% for 1.5–2 min, and finally rinsed twice in sterile distilled water. Four pieces of each sample were placed in each Petri dish containing 2.5% potato dextrose agar (PDA). Dishes were incubated in darkness at 27°C for 7 days and checked every 2 days for the emergence of endophytic fungi. Colonies growing on plates were then transferred to PDA plates. For the biocontrol study, *P. aphanidermatum* strain SQUCC002 was obtained from the Sultan Qaboos University culture collection.

To identity the isolated *Aspergillus* to the species level, total genomic DNA was extracted from freeze dried mycelium using the protocol of Lee and Taylor (1990). The internal transcribed spacer region of the ribosomal RNA (ITS), b-tubulin (TUB) and Calmodulin (CMD) regions were amplified using the primer pairs ITS1 and ITS4 (White et al., 1990), BT2A/BT2B (Koenraadt et al., 1992), and CMD5/CMD6 (Hong et al., 2005), respectively. The temperature profile for the ITS was an initial denaturation step for 10 min at 95°C, followed by 35 cycles of denaturation at 95°C for 30 s, annealing at 55°C for 30 s and extension at 72°C for 90 s and a final extension step of 72°C for 10 min. The temperature profile for TUB and CMD was an initial denaturation step for 5 min at 94°C, followed by 35 cycles of denaturation at 94°C for 45 s, annealing at 55°C for 45 s and extension at 72°C for 60 s and a final extension step of 72°C for 7 min. Purification and sequencing of PCR products were carried out at Macrogen, Korea. Sequences were aligned and improved using MEGA v.6 (Tamura et al., 2013). A maximum likelihood analysis was performed by using raxmlGUI v.1.3 (Silvestro and Michalak, 2012) to identify the species of *Aspergillus* using the combined alignment of ITS, TUB, and CMD regions. The optimal ML tree search was conducted with 1000 separate runs, using the default algorithm. Bootstrap 50% majority-rule consensus trees were generated and the final tree was selected among suboptimal trees from each run by comparing likelihood scores under the GTRGAMMA substitution model. Sequences generated from the analysis were deposited in GenBank under the accession numbers: ITS (65P: MG050978, 9F: MG050979), TUB (65P: MG050980, 9F: MG050981), and CMD: (65P: MG050982, 9F: MG050983).

### Effect of *Aspergillus terreus* on Growth and Morphology of *Pythium aphanidermatum*

The antagonistic activity of *Aspergillus terreus* 65P and 9F isolates was checked against *P. aphanidermatum in vitro*. A 3-mm diameter disk of 2-day old *P. aphanidermatum* culture was placed on the edge of PDA plates. On the opposite edge, a 3-mm diameter disk of 7 day-old *A. terreus* culture was placed. The plates were incubated at 28°C until fungal mycelia of the control plate of *P. aphanidermatum* covered the agar surface. After that, the inhibition zone length (mm) was measured for all plates. The experiment was repeated twice using three replicates each time.

The changes in the hyphal morphology of *P. aphanidermatum* under the effect of *A. terreus* isolates 65P and 9F was screened using a light microscope. The morphology of 50 main hyphae and 50 hyphal tips were thoroughly examined to determine the morphological differences between *P. aphanidermatum* grown on PDA (Control) and *P. aphanidermatum* hypha close to the inhibition zone with *A. terreus*. Three basic morphological characteristics were screened in this study: general shape, internal content (cytoplasm), and end form of both main and hyphal tips. The experiment was repeated twice. Morphology of hyphae was also examined using a scanning electron microscope SEM (INSTUMENT JSM- 5600) at a voltage of 20 kV. A protocol described by Goldstein et al. (2003) was followed to prepare samples for the electron microscopy.

### Effects of *Aspergillus terreus* Culture Filtrate on Electrolyte Leakage and Oospore Production of *Pythium aphanidermatum*

*Aspergillus terreus* isolates 65P and 9F were grown in potato dextrose broth (PDB) for ten days in an incubator at 28°C, and then the fungal mycelium and CF were separated by centrifugation at 10,000 *g*. Then CF was further filtered using Minisart filters with 0.2 μm pore size. The effect of CF of 65P and 9F CFs on *P. aphanidermatum* cellular leakage was studied by measuring extracellular conductivity (Lee et al., 1998; Manhas and Kaur, 2016). *P. aphanidermatum* was grown in PDB for 7 days at 28°C in an incubator shaker. Subsequently the fungal mycelium was obtained by centrifugation for 20 min at 10,000*g*. After that, the mycelium was washed in sterile distilled water and dried on sterile filter paper. Five microgram of dried mycelium was added to 10 ml of 65P and 9F CFs. The supernatants were obtained twice instantly (0 min) and after 24 h of treatment by centrifugation at 10,000*g* for 15 min. Conductivity meter was used to measure the extracellular conductivity for the treatments and control. The test was repeated three times.

The influence of bioactive antifungal metabolites of *A. terreus* strains 65P and 9F on *P. aphanidermatum* oospore production was studied. The CF was mixed with V8 agar at a concentration of 20%, whereas the control plates had V8 agar only. Then the plates were inoculated with 3 mm disk of *P. aphanidermatum* PDA plates at 28°C for three weeks. Oospores were enumerated in 30 consecutive microscopic squares at 40x magnification. There were three replicates for treatments and control.

### Biochemcial Analysis of the Culture Filtrate of *Aspergillus* Isolates

To detect extracellular enzyme (glucanase) production by *A. terreus* isolates the method of Jackson et al. (2013) was followed. Briefly, glucanase substrate (4-MUB-β-D-cellobioside), 4-methylumbelliferone standard, and NaHCO_3_ buffer solutions were prepared. Then the CF of *A. terreus* and the media (PDB) were organized on a 96-well black microplate and mixed with the prepared solutions, using six replicates for each sample. Absorbance was measured at 410 nm, and the following formula was used to determine enzyme activity (Jackson et al., 2013):

Enzyme activity = (mean sample fluorescence – mean initial sample fluorescence)/((mean standard fluorescence/0.5 mol) × (mean quench control fluorescence/mean standard fluorescence) × (0.2 ml) × (time in hour)).

Analysis of *Aspergillus terreus* metabolites was done by growing *A. terreus* isolates 65P and 9F in PDB for ten days in an incubator at 28°C. Then the CF was separated from fungal mycelium spores in a centrifuge, followed by filtration through Minisart filters with 0.2 μm pore size. Liquid chromatography–mass spectrometry (LC-MS) was used for the detection of metabolites produced by *A. terreus* isolates depending on their masses. The CFs of *A. terreus* isolates 65P and 9F were concentrated by a freeze dryer machine to 25% of its total volume, then injected directly to LC-MS equipped with an electrospray ionization (ESI) source. The analysis was performed in positive polarities at ion spray voltage of +3000 V, Frag = 5.0V CF = 0.000, and DF = 0.000.

Four media were prepared to detect siderophore production by 65P and 9F strains including: King B medium consisting of glycerine 10 g/L, proteose-peptone 20 g/L, and MgSO4 1.5 g/L (de Villegas et al., 2002), glucose medium consisting of K_2_HPO_4_ 0.56 g/L, Glucose 10 g/L, urea 0.85 g/L, and Glutamic acid 1 g/L (de Villegas et al., 2002), citrate medium consisting of K_2_HP0_4_ 6.0 g/L, KH2P04 3.0 g/L, (NH_4_)2S0_4_ 1.0 g/L, MgS0_4_.7H_2_0 0.2 g/L, and citric acid 4.0 g/L (Meyer and Abdallah, 1978) and asparagine medium consisting of Asparagine 5 g/L, MgSO_4_ 0.1 g/L, and K_2_HPO_4_ 0.5 g/L (Rachid and Ahmed, 2005). The pH for all the media was adjusted to 7.0. A 3 mm diameter mycelial disk taken from fresh potato dextrose agar plates of 65P and 9F isolates was transferred to the prepared media and incubated at 28°C on a rotary shaker (120 rpm) for 5 days. The culture broths were centrifuged at 10,000 *g* for 15 min and the supernatants were filtered through 0.2 μM Minisart filters. The absorbance of the supernatants was measured at 400 nm using six replicates for each sample.

The concentration was calculated according to method of Meyer and Abdallah (1978) using absorption maximum (λ = 400 nm) and molar extinction coefficient ε = 20000 (Rachid and Ahmed, 2005).

### Control of *Pythium*-Induced Damping-Off of Cucumber by *Aspergillus* Isolates

The effect of 65P and 9F *A. terreus* isolates on Pythium damping-off of cucumber was studied using the following experimental approach. Four pots (12-cm in diameter) were used, with seven cucumber seeds sown in each pot. There were one control (irrigated with PDB) and three treatments: the first treatment was inoculated with a 57 mm plate of *P. aphanidermatum*, 2 cm below soil level; the second treatment was irrigated with 25 ml spore/mycelial suspension of 65P or 9F *A. terreus* isolates and the third treatment was inoculated with *P. aphanidermatum* and irrigated with 25ml spore/mycelial suspension of 65P or 9F *A. terreus* isolates (Al-Hinai et al., 2010). Pots were incubated in a glasshouse and the temperature was adjusted at 28°C. After 3 weeks the surviving seedlings, seedlings shoot length, seedlings fresh weight and seedlings dry weight were determined. The experiment was repeated twice.

### Statistical Analysis

Data were analyzed using IBM SPSS Statistics 24.0. Treatment means ± SD were compared using independent sample *t*-test, One-way ANOVA and Duncan’s Multiple Range Test. Pearson Chi-Square test was used for *P. aphanidermatum* morphological study and Poisson test was applied for spore production count analyze.

## Results

### Identification of *Aspergillus* Isolates

**FIGURE 1:**
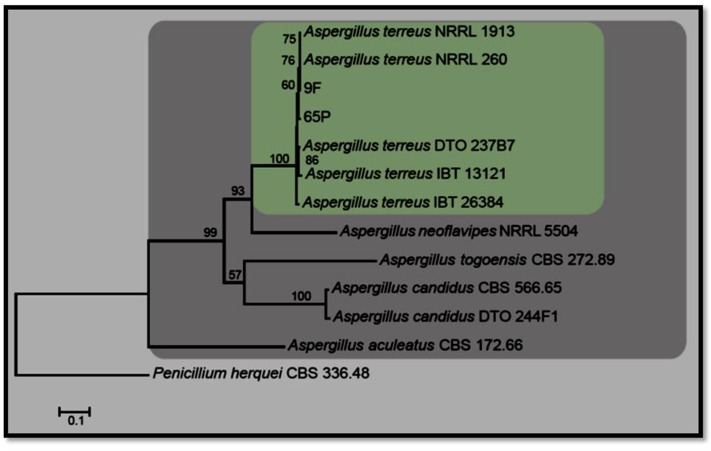
RaXML tree for the analysis *Aspergillus* spp. based on the combined ITS, TUB, and CMD gene regions. The tree is rooted with *Penicillium herquei* (CBS 336.48).

*Aspergillus* isolate 65P was isolated from the root of *R. stricta* and *Aspergillus* isolate 9F was isolated from the root of *T. apollinea*. Species in *Aspergillus* are shown in **Figure 1**. The combined ITS, TUB and CMD dataset comprises 12 isolates of *Aspergillus* with *Penicillium herquei* (CBS 336.48) as the outgroup taxon. The manually adjusted dataset comprised 1664 characters including gaps (ITS: 1-565, TUB: 566-1102, CMD: 1103- 1664). A best scoring RAxML tree resulted with the value of Likelihood: –7589.972036 (**Figure 1**). The *Aspergillus* isolates in this study grouped with previously published *A. terreus* with high a boostrap support.

### Effect of *Aspergillus terreus* on Growth and Morphology of *Pythium aphanidermatum*

The antagonism test showed the suppression of *P. aphanidermatum* growth in PDA plates under the influence of *A. terreus* isolates. This suppression was illustrated through the production of an inhibition zone by *A. terreus*. The inhibition zone produced by isolate 65P (8.66 mm) was significantly larger than the one produced by isolate 9F (**Table 1**).

**Table 1:** Effect of *Aspergillus terreus* isolates 65P and 9F on the inhibition of *Pythium aphanidermatum* growth and the effect of their culture filtrate (CF) on extracellular conductivity (0–24 h) and spore production by *P. aphanidermatum.*

	Inhibition zone (mm)	Extracellular conductivity (mV)	Spore production (no.)
Control	0 c	2.1 b	73 a
9F	5.8 b	24.6 a	17 b
65P	8.7 a	35.7 a	18 b

*Values with the same letters in the same column are not significantly different from each other (Duncan test, *P* > 0.05)*.

**FIGURE 2:**
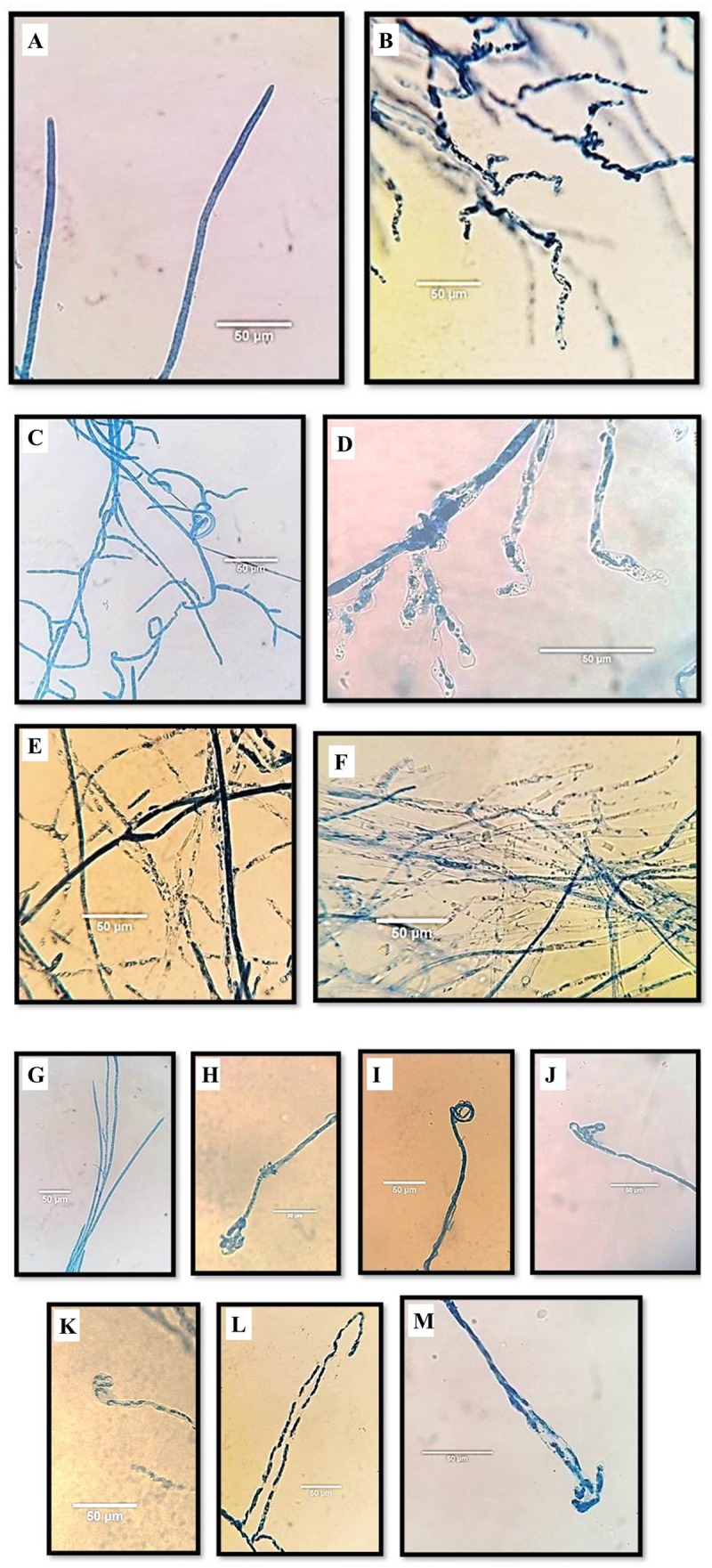
Effect of *Aspergillus terreus* 65P and 9F isolates on *Pythium aphanidermatum* morphology using light microscope. Normal general shape in control plate **(A)** vs. disintegrated/wavy hyphae **(B)**; **(C)** the normal internal content; **(D)** abnormal hyphae: semi-empty pattern with content degradation under influence of 9F treatment, and abnormal hyphae: pale granular, semi empty and empty under influence of 65P **(E)** and 9F **(F)** treatments consecutively; **(G)** normal ends in control plate (no treatment); **(H,I)** wrapped up end under effect 65P treatment; **(J)** wrapped up end under effect 9F treatment; **(K)** swollen end under effect 9F treatment and **(L,M)** hook-like end under effect of 65P and 9F treatments, consecutively.

**FIGURE 3:**
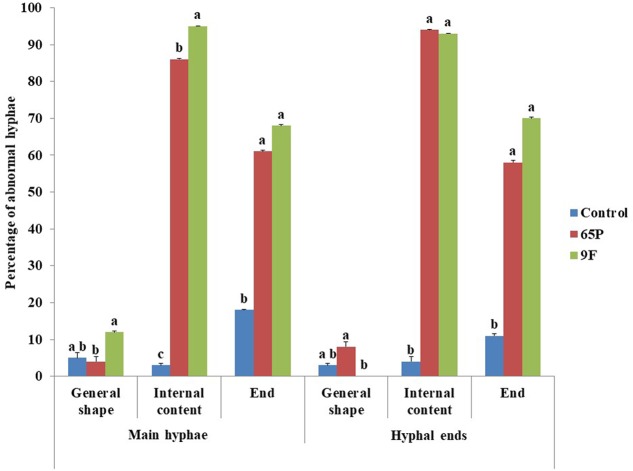
Effect of *A. terreus* on the hyphal morphology of *P. aphanidermatum*. Columns and bars represent percentage ± CV of SD. Values with the same letters are not significantly different from each other (Pearson Chi-Square: asymptotic significance, 2-sided; > 0.05).

Microscopic examination showed that *Aspergillus* isolates 65P and 9F induced significant changes in the general appearance, content and ends of *P. aphanidermatum* hyphae (**Figure 2**). The effects were on the main hyphae and hypha branches close to the inhibition zone. The general appearance of hyphae became wavy (**Figure 3**), while for the internal content the hyphae lost most or part of its content (the cytoplasm) (**Figure 3**). Hyphal ends were also affected (**Figure 3**). Significant differences were found between the two *Aspergillus* isolates in their effect on general shape of main hypha and branches and also on the internal content of the main hyphae.

**FIGURE 4:**
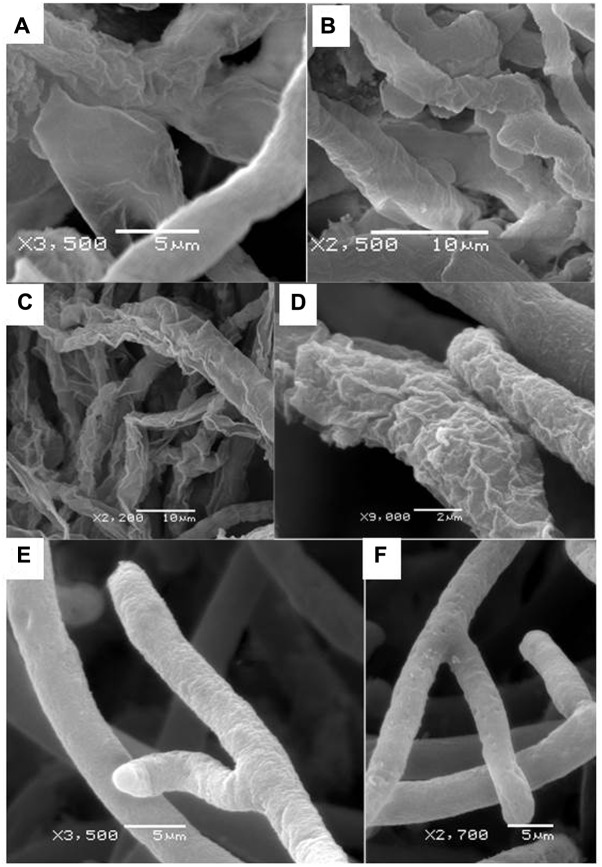
Effect of *A. terreus* on the *P. aphanidermatum* hyphae morphology using SEM. **(A,B)** Abnormal hyphae: wrinkled or shrunken patterns under effect of 9F treatment **(C,D)** abnormal hyphae: wrinkled or shrunken patterns with under effect of 65P treatment, **(E,F)** normal patterns of hyphae in the control.

Furthermore, the scanning electron microscope showed that *Aspergillus* isolates 65P and 9F caused considerable changes in *P. aphanidermatum* hyphal morphology (**Figure 4**). Most of the observed hyphal patterns were wrinkled in both treatments compared to control which had normal hyphae.

### Effects of *Aspergillus terreus* Culture Filtrate on Electrolyte Leakage and Oospore Production of *Pythium aphanidermatum*

Treating *P. aphanidermatum* with the CF of *Aspergillus* isolates 65P and 9F resulted in significantly higher extracellular conductivity values after 24 h in comparison with the control, with the values been 35.7 mV for 65P, 24.6 mV for 9F and 2.1 mV for the control (**Table 1**). This gives indication that the mycelium of *P. aphanidermatum* leaked electrolytes as a result of possible enzymes or metabolites produced by *A. terreus*.

The CFs of 65P and 9F isolates of *A. terreus* significantly reduced spore production by *P. aphanidermatum* compared to the control (*P* < 0.05) (**Table 1**). Oospores which were produced in control (73) were higher than the spores in 65P treatment (18) and 9F treatment (17).

### Biochemical Analysis of the Culture Filtrate of *Aspergillus* Isolates

The concentration of glucanase in the CFs of 65P (3.99) and 9F (4.73) were significantly higher than the media control which was (0.47). Enzyme activity is expressed in nmoles substrate consumed h^-1^ ml of CF or media^-1^). This confrms that *A. terreus* isolaetes produce glucanse enzyme and its activity increases within minutes of application.

The spectra of isolate 65P CF showed a set of peaks in the positive and negative ion *ESI* spectra. The positive spectra showed distributions of peaks ranging between 146.8000 and 909.6000 m/z (**Supplementary Figure S1**). However, peaks in the negative ion mode spectra were in the range of 148.8000 to 2522.7000 m/z. Peaks in the positive and negative ion mode spectra for isolate 9F were in the ranges of 132.0000 to 928.5000 m/z and 149.0000 to 2560.3000 m/z, respectively (**Supplementary Figure S2**).

Siderophore production by 65P and 9F isolates in four media is displayed in **Table 2**. It indicates siderophore secretion by both isolates of *A. terreus* at varying values depending on the medium. The best medium that contains the highest amount of siderphore was King B medium for 65P isolate (93.63 μM) and 9F isolate (55.88 μM), followed by Glucose medium (63.78 μM for 65P isolate and 15.91 μM for 9F isolate). However, Citrate and Asparagine media had low amounts of siderophore compared to King B and Glucose media.

**Table 2:** Effect of medium content on siderophores concentration (μM) produced by 65P and 9F isolates.

Medium	King B medium	Glucose medium	Citrate medium	Asparagine medium
65P	93.63^a^ ± 3.74	63.78^b^ ± 3.23	7.18^e^ ± 0.11	6.96^e^ ± 0.32
9F	55.88^c^ ± 1.29	15.91^d^ ± 0.52	6.99^e^ ± 0.11	7.20^e^ ± 0.1

*Values (concentration in μM ± SD) with the same letters are not significantly different from each other (Duncan test, *P* > 0.05)*.

### Control of *Pythium*-Induced Damping-Off of Cucumber by *Aspergillus* Isolates

**FIGURE 5:**
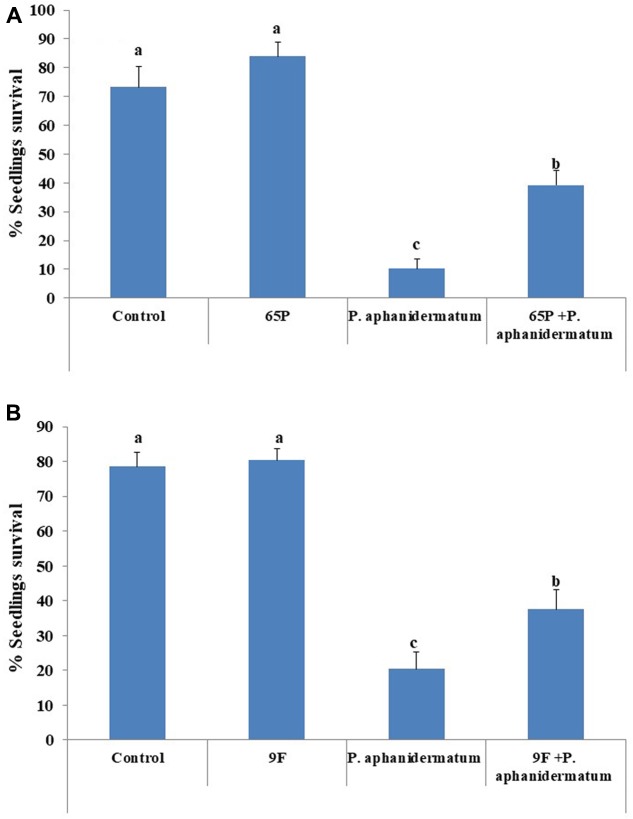
The biocontrol efficacy of endophytic fungi treatments against *P. aphanidermatum* damping-off. **(A)** 65P treatment **(B)** 9F treatment. Columns and bars represent percentage ±95% confidence limit. Values with the same letters are not significantly different from each other (Duncan test, *P* > 0.05).

Inoculation of cucumber seedlings with *P. aphanidermatum* resulted in the development of damping-off symptoms, with only 10–20% of the seedlings survived. The rate of seedling survival increased to 39.3% in seedlings treated by 65P and 37.5% in seedlings treated by 9F (**Figure 5**).

**FIGURE 6:**
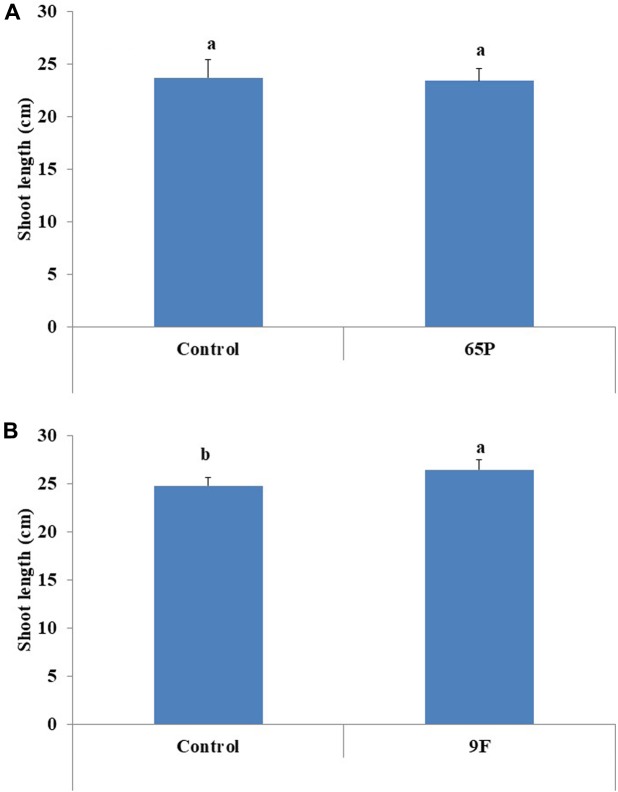
Effect of inoculation with 65P **(A)** and 9F **(B)** fungi on cucumber seedlings shoot length. Error bars represent 95% confidence limit of a means. Values with the same letters are not significantly different from each other (ANOVA Test, *P* > 0.05).

**FIGURE 7:**
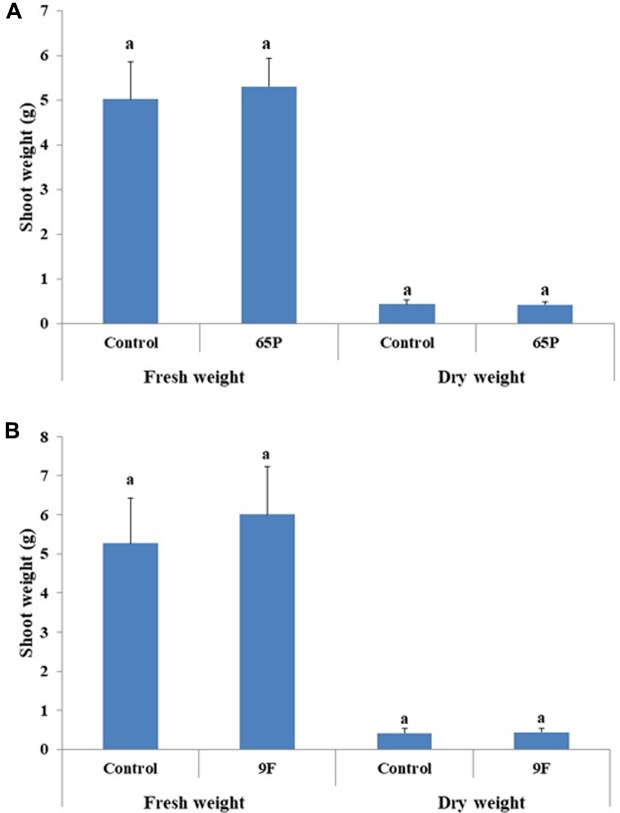
Effect of inoculation with 65P **(A)** and 9F **(B)** fungi on cucumber seedlings shoot weight. Error bars represent 95% confidence limit of a means. The same letters are not significantly different from each other (ANOVA Test, *P* > 0.05).

No significant effect was found for the 65P isolate on the shoot length of cucumber seedlings, while the 9F isolate significantly increased the shoot length of cucumber seedlings (**Figure 6**). No effect was found for *Aspergillus* isolates on the shoot fresh and dry weight (**Figure 7**).

## Discussion

The current study identified *Aspergillus terreus* as an entophytic fungus in *R. stricta* and *T. apollinea*. *A. terreus* was not isolated previously from *R. stricta* and *T. apollinea*. *A. terreus* was identified to the species level based on sequences of the ITS, TUB, and CALMODULIN genes, which were efficient in discriminating this species from other *Aspergillus* species (Peterson, 2008; Arabatzis and Velegraki, 2013; Samson et al., 2014).

*Aspergillus terreus* 65P and 9F isolates inhibited the growth of *P. aphanidermatum in vitro*. A study carried out by Kumar et al. (2000) showed the production of an inhibition zone by *A. terreus* against *Neurospora crassa*. Moreover, a study by Wang et al. (2011) revealed that new compounds from *A. terreus* could produce inhibition zone against *Pseudomonas aeruginosa* and *Enterobacter aerogenes* growth. It is likely that an inhibition zone was produced because *A. terreus* secrets metabolites that interfere with the growth of *P. aphanidermatum*. Ferrón et al. (2005) and Wang et al. (2011) reported the production of terremides A and B and lovastatin by *A. terreus* that were found responsible for the production of inhibition zones. In our study, the use of CF from *A. terreus* isolates resulted in electrolyte leakage in *P. aphanidermatum* mycelium and also interfered with oospore production in *P. aphanidermatum*. Extracellular conductivity of *Streptomyces hydrogenans* supernatant that was treated with *Alternaria brassicicola* mycelium was increased with the progress of time compared to 0 min (Manhas and Kaur, 2016). *P. aphanidermatum* cell wall polysaccharides consist of 18% of cellulose and 82% of (1 → 3), (1 → 6)-β-D-glucans (Blaschek et al., 1992). Our study showed that *A. terreus* 65P and 9F isolates have the ability to produce glucanase enzyme. A previous study by Gao et al. (2008) proved the production of extracellular enzymes by thermoacidophilic fungal *A. terreus* M11. Also, the results of Djonović et al. (2006) demonstrated the involvement of β-1,6-glucanase in mycoparasitism and its relevance in the biocontrol activity of *Trichoderma virens* against plant pathogen *Pythium ultimum*. Furthermore, *T. harzianum* produced 1,3-β-glucanase and cellulase which led to control Pythium damping-off of cucumber seedlings (Thrane et al., 1997). In addition, El-Tarabily et al. (2009) showed that the glucanase-producing actinomycetes could replace the use of metalaxyl in the control of *Pythium aphanidermatum* diseases. Many other fungi have the ability to produce extracellular enzymes such as *Phoma medicaginis* and *Penicillium citrinum* (Khan et al., 2016) and *Talaromyces emersonii* (McHale and Coughlan, 1981). The production of extracellular enzymes by *Talaromyces flavus* effectively contributed to control of *Sclerotium rolfsii* and *Verticillium dahliae* (Madi et al., 1997). The electrolyte leakage, which was observed in our study, appears to be because of the glucanase enzyme production by *A. terreus* isolates, which appears to play a role in the biological control against *P. aphanidermatum* through the degradation of *Pythium* cell wall and release of cell components.

*Aspergillus terreus* has been shown to produce several metabolites (Nakagawa et al., 1982; Hendrickson et al., 1999; Gao et al., 2008; Goutam et al., 2017; Saha et al., 2017). In our study, by comparing the current molecular weight results of 65P (389.1 m/z) and 9F (390.7 m/z) with the findings from previous studies, both isolates seem to produce mevastatin (390.513 g/mol). Mevastatin production by *A. terreus* was reported by previous studies (Manzoni et al., 1998). It has also been reported to be produced by other fungi (Brown et al., 1976; Reino et al., 2008). Mevastatin has been reported to have biological inhibitory activities (Kumar et al., 2000). Thus, it is possible that the production of this compound and others could explain the suppression role of *A. terreus* isolates against *P. aphanidermatum.*

Our study showed that *A. terreus* isolates induced morphological changes in the mycelium of *P. aphanidermatum*. The general shape of *Pythium* mycelium changed from straight normal pattern to abnormal patterns as wavy, and the internal content became granular, disintegrated and lost. A previous study by Chet et al. (1981) showed that *Trichoderma hamatum* induced morphological changes in *Pythium* spp and *Rhizoctonia solani*, where the mycelium had bulbular or hook-like structures that contained granular cytoplasm. Another study by Paulitz et al. (2000) showed that *Pseudomonas aureofaciens* resulted in abnormalities in hyphal morphology of *Pythium ultimum*. Prapagdee et al. (2008) showed that *Streptomyces hygroscopicus* produced extracellular antifungal metabolites such as chitinase and β-1,3-glucanase that affected the growth and morphology of *Colletotrichum gloeosporioides* and *Sclerotium rolfsii* phytopathogenic fungi. Several metabolites such as enzymes, antibiotics and organic acids are secreted by *A. terreus* (Calton et al., 1978; Nakagawa et al., 1982; Hendrickson et al., 1999; Gao et al., 2008; Goutam et al., 2017; Saha et al., 2017; Sreedevi et al., 2017). In our study, it is very likely that the production of glucanase enzyme contributed to abnormalities in *Pythium* mycelia.

Our scanning electron microscope results showed that *P. aphanidermatum* hyphae became wrinkled or shrunken as well as smaller in size; however, in control the hyphae had normal cell wall morphology with a smooth surface and full content. These results indicate that *P. aphanidermatum* cytoplasm in the treated petri dishes plates were degraded by the effect of *A. terreus* isolates and their enzyme, glucanase. This shrunken morphology was observed in *Fusarium oxysporum* mycelia which were treated with *Streptomyces cinereus* (Gangwar et al., 2015).

Spore production by *P. aphanidermatum* has been inhibited by the CF of 65P and 9F isolates. A previous study by El-Tarabily (2006) showed that *P. aphanidermatum* oospores were parasitized by *Actinoplanes philippinensis* and *Micromonospora chalcea* and as a result had disorganized cytoplasm. Also, Manhas and Kaur (2016) study demonstrated suppression of germination as well as loss of pigmentation and shrinkage of *Alternaria brassicicola* spores due to treatment with the CF of *Streptomyces hydrogenans*. Only *Actinoplanes campanulatus* was capable of affecting *P. aphanidermatum* oospores in El-Tarabily et al. (2009) study.

Our results proved that both *A. terreus* isolates can produce siderophore, with some differences between the isolates in their ability to produce siderophore. King B was the best medium for obtaining the highest value of siderophore. Similarly Rachid and Ahmed (2005) found that king B medium is better than several other media. Many studies indicated siderophore production by several fungi such as *Aspergillus terreus* (Waqas et al., 2015), *Aspergillus fumigatus* (Haas, 2014), and *Chaetostylum fresenii* (Thieken and Winkelmann, 1992).

Siderophore production is an effective mechanism of biological control of multiple diseases caused bacterial and fungal agents. Siderophore production by *Rhodotorula glutinis* has been reported to suppress blue rot caused by *Penicillium expansum* in harvested apples (Calvente et al., 1999). Moreover, *Bacillus subtilis* inhibits Fusarium-wilt of tomatoes disease and secrets siderophore. Furthermore, the inhibition role of *Pseudomonas fluorescens* against several *Pythium* species is due to siderophore production (Weller and Cook, 1986).

The bioassay test showed a significant effect of *A. terreus* on the survival of cucumber seedlings inoculated by *P. aphanidermatum*. The *A. terreus* isolates did not have any negative effects on the shoot length, dry weight, or fresh weight. Various studies were carried out to characterize the biocontrol efficacy against *P. aphanidermatum* damping-off by multiple microorganisms such as *Trichoderma harzianum* (Sivan et al., 1984), *Pseudomonas chlororaphis*, and *Bacillus subtilis* (Nakkeeran et al., 2006) and endophytic actinomycetes (El-Tarabily et al., 2009). Some studies used several biocontrol species, which led to greater disease suppression (Szczech and Shoda, 2004; Liu et al., 2017). Other studies used seed treatment with biocontrol agents which proved its effectiveness in damping-off control (Lifshitz et al., 1986; Callan et al., 1990).

Our study demonstrates for the first time that *A. terreus* can be effectively used to manage *Pythium*-induced damping-off of cucumber. *A. terreus* was found to interfere with the growth and spore production of *P. aphanidermatum* and induce morphological changes in its mycelium. In addition, *A. terreus* produces glucanase enzyme and mevastatin, the activities of which are suggested to play a role in the antagonism against *P. aphanidermatum*. In addition to the active role of enzymes in biological control, several other secondary metabolites contribute effectively to the control of plant pathogens such as siderophore (Schwyn and Neilands, 1987; Naureen et al., 2017) and Hydrogen cyanide (HCN) (Ramette et al., 2003). Baakza et al. (2004) indicated production of siderophore by *Aspergillus* species including *A. terreus*. It is therefore important to conduct a separate study to look into all the possible mechanisms by which *A. terreus* inhibits or interfere with *P. aphanidermatum*. Since *A. terreus* is known to have some side effects on human health, future studies should take into account investigating the side effects, if any, of *A. terreus* when used as a biocontrol agent. These include effects of plants as well as safety during applications. The effects of *A. terreus* on other biocontrol agents in soil should also be investigated.

## Author Contributions

BH, RA-Y, and AA-S designed the experiment. BH carried out the experiments. BH and AA-S analyzed the data. BH, RA-Y, and AA-S wrote the manuscript. All authors approved the manuscript.

## Conflict of Interest Statement

The authors declare that the research was conducted in the absence of any commercial or financial relationships that could be construed as a potential conflict of interest.

## Supplementary Material

FIGURE S1 Mass spectrum (m/z) of *Aspergillus terreus* 65P isolate culture filtrate (CF). **(A–C)** Shows positive ESI scan (at 0.146 min and 100–3000 m/z), (at 156 min and 100:1075 m/z), (at 156 min and 260–920 m/z), respectively, while **(D–F)** show negative ESI scan (at 0.146 min and 100:3000 m/z), (at 0.146 min and 100:1250 m/z), (at 0.146 min and 1050:3000 m/z), respectively.

FIGURE S2 Mass spectrum (m/z) of *A. terreus* 9F isolate CF. **(A–C)** Shows positive ESI scan (at 0.146 min and 100–3000 m/z), (at 136 min and 100:1125 m/z), (at 136 min and 280–1140 m/z), respectively, while **(D–F)** show negative ESI scan (at 0.146 min and 100:3000 m/z), (at 0.146 min and 100:1300 m/z), (at 0.146 min and 500:3000 m/z), respectively.

## References

[B1] Abdallah R. A.-B. Hassine M. Jabnoun-Khiareddine H. Haouala R. Daami-Remadi M. (2014). Antifungal activity of culture filtrates and organic extracts of Aspergillus spp. against *Pythium ultimum. Tunis. J. Plant Prot.* 9 17–30.

[B2] Abdelzaher H. M. A. Gherbawy Y. A. M. H. Elnaghy M. A. (2000). Damping-off disease of maize caused by *Pythium deliense* meurs in El-Minia, Egypt and its possible control by some antagonistic soil fungi. *Egypt. J. Microbiol.* 35 21–45.

[B3] Al-Hinai A. H. Al-Sadi A. M. Al-Bahry S. N. Mothershaw A. S. Al-Said F. A. Al-Harthi S. A. (2010). Isolation and characterization of *Pseudomonas aeruginosa* with antagonistic activity against *Pythium aphanidermatum*. *J. Plant Pathol.* 92 653–660.

[B4] Al-Kiyumi K. S. (2006). *Greenhouse Cucumber Production Systems in Oman: A Study on the Effect of Cultivation Practices on Crop Diseases and Crop Yields.* Reading: University of Reading.

[B5] Al-Sadi A. M. Al-Ghaithi A. G. Al-Balushi Z. M. Al-Jabri A. H. (2012). Analysis of diversity in *Pythium aphanidermatum* populations from a single greenhouse reveals phenotypic and genotypic changes over 2006 to 2011. *Plant Dis.* 96 852–858. 10.1094/pdis-07-11-0624 30727347

[B6] Al-Sadi A. M. Al-Masoodi R. S. Al-Ismaili M. Al-Mahmooli I. H. (2015a). Population structure and development of resistance to hymexazol among *Fusarium solani* populations from date palm, citrus and cucumber. *J. Phytopathol.* 163 947–955. 10.1111/jph.12397

[B7] Al-Sadi A. M. Al-Masoudi R. S. Al-Habsi N. Al-Said F. A. Al-Rawahy S. A. Ahmed M. (2010). Effect of salinity on Pythium damping-off of cucumber and on the tolerance of *Pythium aphanidermatum*. *Plant Pathol.* 59 112–120. 10.1111/j.1365-3059.2009.02176.x

[B8] Al-Sadi A. M. Al-Oweisi F. A. Edwards S. G. Al-Nadabi H. Al-Fahdi A. M. (2015b). Genetic analysis reveals diversity and genetic relationship among *Trichoderma* isolates from potting media, cultivated soil and uncultivated soil. *BMC Microbiol.* 15:147. 10.1186/s12866-015-0483-8 26215423 PMC4517564

[B9] Al-Sadi A. M. Al-Said F. A. Al-Kiyumi K. S. Al-Mahrouqi R. S. Al-Mahmooli I. H. Deadman M. L. (2011). Etiology and characterization of cucumber vine decline in Oman. *Crop Prot.* 30 192–197. 10.1016/j.cropro.2010.10.013

[B10] Al-Sa’di A. Drenth A. Deadman M. Aitken E. (2008). Genetic diversity, aggressiveness and metalaxyl sensitivity of *Pythium aphanidermatum* populations infecting cucumber in Oman. *Plant Pathol.* 57 45–56.

[B11] Arabatzis M. Velegraki A. (2013). Sexual reproduction in the opportunistic human pathogen *Aspergillus terreus*. *Mycologia* 105 71–79. 10.3852/11-426 23074177

[B12] Asaka O. Shoda M. (1996). Biocontrol of *Rhizoctonia solani* damping-off of tomato with *Bacillus subtilis* RB14. *Appl. Environ. Microbiol.* 62 4081–4085. 16535440 10.1128/aem.62.11.4081-4085.1996 PMC1388978

[B13] Baakza A. Dave B. Dube H. (2004). Chemical nature, ligand denticity and quantification of fungal siderophores. *Indian J. Exp. Biol.* 42 96–105. 15274489

[B14] Berg L. E. Miller S. S. Dornbusch M. R. Samac D. A. (2017). Seed rot and damping-off of alfalfa in Minnesota caused by Pythium and Fusarium species. *Plant Dis.* 101 1860–1867. 10.1094/PDIS-02-17-0185-RE 30677318

[B15] Blaschek W. Käsbauer J. Kraus J. Franz G. (1992). *Pythium aphanidermatum*: culture, cell-wall composition, and isolation and structure of antitumour storage and solubilised cell-wall (1 → 3),(1 → 6)-β-d-glucans. *Carbohydr. Res.* 231 293–307. 10.1016/0008-6215(92)84026-O 1394320

[B16] Brown A. G. Smale T. C. King T. J. Hasenkamp R. Thompson R. H. (1976). Crystal and molecular structure of compactin, a new antifungal metabolite from *Penicillium brevicompactum*. *J. Chem. Soc. Perkin* 1 1165–1170. 10.1039/p19760001165 945291

[B17] Burgess K. M. Ibrahim A. Sørensen D. Sumarah M. W. (2017). Trienylfuranol A and trienylfuranone A–B: metabolites isolated from an endophytic fungus, Hypoxylon submoniticulosum, in the raspberry *Rubus idaeus*. *J. Antibiot.* 70 721–725. 10.1038/ja.2017.18 28246381

[B18] Callan N. W. Mathre D. Miller J. B. (1990). Bio-priming seed treatment for biological control of *Pythium ultimum* preemergence damping-off in sh-2 sweet corn. *Plant Dis.* 74 368–372. 10.1094/PD-74-0368

[B19] Calton G. Ranieri R. Espenshade M. (1978). Quadrone, a new antitumor substance produced by *Aspergillus terreus*. *J. Antibiot.* 31 38–42. 10.7164/antibiotics.31.38 627521

[B20] Calvente V. Benuzzi D. de Tosetti M. S. (1999). Antagonistic action of siderophores from *Rhodotorula glutinis* upon the postharvest pathogen *Penicillium expansum*. *Int. Biodeter. Biodegrad.* 43 167–172. 10.1016/S0964-8305(99)00046-3

[B21] Chet I. Harman G. Baker R. (1981). *Trichoderma hamatum*: Its hyphal interactions with *Rhizoctonia solani* and Pythium spp. *Microb. Ecol.* 7 29–38. 10.1007/BF02010476 24227317

[B22] de Villegas M. D. Villa P. Frias A. (2002). Evaluation of the siderophores production by *Pseudomonas aeruginosa* PSS. *Rev. Latinoam. Microbiol.* 44 112–117. 17061484

[B23] Deadman M. Al Maqbali Y. Al Sa’di A. Al Hasani H. Al Nabhani M. (2007). Biofumigation for the management of damping-off in greenhouse cucumbers in the sultanate of Oman. *Acta Hortic.* 731 367–370. 10.17660/ActaHortic.2007.731.49

[B24] Djonović S. Pozo M. J. Kenerley C. M. (2006). Tvbgn3, a β-1, 6-glucanase from the biocontrol fungus *Trichoderma virens*, is involved in mycoparasitism and control of *Pythium ultimum*. *Appl. Environ. Microbiol.* 72 7661–7670. 10.1128/AEM.01607-06 16997978 PMC1694269

[B25] El-Tarabily K. A. (2006). Rhizosphere-competent isolates of streptomycete and non-streptomycete actinomycetes capable of producing cell-wall-degrading enzymes to control *Pythium aphanidermatum* damping-off disease of cucumber. *Botany* 84 211–222. 10.1139/b05-153

[B26] El-Tarabily K. Nassar A. Hardy G. S. J. Sivasithamparam K. (2009). Plant growth promotion and biological control of *Pythium aphanidermatum*, a pathogen of cucumber, by endophytic actinomycetes. *J. Appl. Microbiol.* 106 13–26. 10.1111/j.1365-2672.2008.03926.x 19120624

[B27] Ferrón M. V. López J. C. Pérez J. S. Sevilla J. F. Chisti Y. (2005). Rapid screening of *Aspergillus terreus* mutants for overproduction of lovastatin. *World J. Microbiol. Biotechnol.* 21 123–125. 10.1007/s11274-004-3045-z

[B28] Gangwar M. Kaur N. Saini P. Kalia A. (2015). The diversity, plant growth promoting and anti-microbial activities of endophytic actinomycetes isolated from Emblica officinalis Gaertn. *Int. J. Adv. Res.* 3 1062–1071.

[B29] Gao J. Weng H. Zhu D. Yuan M. Guan F. Xi Y. (2008). Production and characterization of cellulolytic enzymes from the thermoacidophilic fungal *Aspergillus terreus* M11 under solid-state cultivation of corn stover. *Bioresour. Technol.* 99 7623–7629. 10.1016/j.biortech.2008.02.005 18346891

[B30] Georgakopoulos D. G. Fiddaman P. Leifert C. Malathrakis N. E. (2002). Biological control of cucumber and sugar beet damping-off caused by *Pythium ultimum* with bacterial and fungal antagonists. *J. Appl. Microbiol.* 92 1078–1086. 10.1046/j.1365-2672.2002.01658.x 12010548

[B31] Goldstein J. I. Newbury D. E. Echlin P. Joy D. C. Lyman C. E. Lifshin E. (eds) (2003). “Special topics in scanning electron microscopy,” in *Scanning Electron Microscopy and X-Ray Microanalysis* (Berlin: Springer), 195–270.

[B32] Goudjal Y. Toumatia O. Yekkour A. Sabaou N. Mathieu F. Zitouni A. (2014). Biocontrol of *Rhizoctonia solani* damping-off and promotion of tomato plant growth by endophytic actinomycetes isolated from native plants of Algerian Sahara. *Microbiol. Res.* 169 59–65. 10.1016/j.micres.2013.06.014 23920229

[B33] Goutam J. Sharma G. Tiwari V. K. Mishra A. Kharwar R. N. Ramaraj V. (2017). Isolation and characterization of “Terrein” an antimicrobial and antitumor compound from endophytic fungus *Aspergillus terreus* (JAS-2) associated from *Achyranthes aspera* Varanasi, India. *Front. Microbiol.* 8:1334 10.3389/fmicb.2017.01334 PMC5526331 28790982

[B34] Haas H. (2014). Fungal siderophore metabolism with a focus on *Aspergillus fumigatus*. *Nat. Prod. Rep.* 31 1266–1276. 10.1039/c4np00071d 25140791 PMC4162504

[B35] Hatami N. Aminaee M. M. Zohdi H. Tanideh T. (2013). Damping-off disease in greenhouse cucumber in Iran. *Arch. Phytopathol. Plant Prot.* 46 796–802. 10.1080/03235408.2012.752145

[B36] Hendrickson L. Davis C. R. Roach C. Aldrich T. McAda P. C. Reeves C. D. (1999). Lovastatin biosynthesis in *Aspergillus terreus*: characterization of blocked mutants, enzyme activities and a multifunctional polyketide synthase gene. *Chem. Biol.* 6 429–439. 10.1016/S1074-5521(99)80061-1 10381407

[B37] Hong S.-B. Go S.-J. Shin H.-D. Frisvad J. C. Samson R. A. (2005). Polyphasic taxonomy of *Aspergillus fumigatus* and related species. *Mycologia* 97 1316–1329. 10.1080/15572536.2006.11832738 16722222

[B38] Howell C. (2003). Mechanisms employed by Trichoderma species in the biological control of plant diseases: the history and evolution of current concepts. *Plant Dis.* 87 4–10. 10.1094/PDIS.2003.87.1.4 30812698

[B39] Hrunyk N. Gout R. Kovaleva V. (2017). Regulation of gene expression for defensins and lipid transfer protein in Scots pine seedlings by necrotrophic pathogen *Alternaria alternata* (Fr.). *Folia For. Pol.* 59 152–158. 10.1515/ffp-2017-0015

[B40] Jackson C. R. Tyler H. L. Millar J. J. (2013). Determination of microbial extracellular enzyme activity in waters, soils, and sediments using high throughput microplate assays. *J. Vis. Exp.* 80:e50399. 10.3791/50399 24121617 PMC3938205

[B41] Khan A. L. Al-Harrasi A. Al-Rawahi A. Al-Farsi Z. Al-Mamari A. Waqas M. (2016). Endophytic fungi from frankincense tree improves host growth and produces extracellular enzymes and indole acetic acid. *PLOS ONE* 11:e0158207. 10.1371/journal.pone.0158207 27359330 PMC4928835

[B42] Khan R. Shahzad S. Choudhary M. I. Khan S. A. Ahmad A. (2010). Communities of endophytic fungi in medicinal plant *Withania somnifera*. *Pak. J. Bot.* 42 1281–1287.

[B43] Kim K.-K. Kang J.-G. Choi Y.-L. Yun H.-D. Ha H.-S. Kang K.-Y. (1998). Characterization of an antifungal compound isolated from an antagonistic fungus *Aspergillus terreus* against phytopathogenic fungi. *Korean J. Pestic. Sci.* 2 40–45.

[B44] Koenraadt H. Somerville S. C. Jones A. (1992). Characterization of mutations in the beta-tubulin gene of benomyl-resistant field strains of *Venturia inaequalis* and other plant pathogenic fungi. *Phytopathology* 82 1348–1354. 10.1094/Phyto-82-1348

[B45] Kraus J. Loper J. E. (1992). Lack of evidence for a role of antifungal metabolite production by *Pseudomonas fluorescens* Pf-5 in biological control of Pythium damping-off of cucumber. *Phytopathology* 82 264–271. 10.1094/Phyto-82-264

[B46] Kumar M. S. Kumar P. M. Sarnaik H. M. Sadhukhan A. (2000). A rapid technique for screening of lovastatin-producing strains of *Aspergillus terreus* by agar plug and *Neurospora crassa* bioassay. *J. Microbiol. Methods* 40 99–104. 10.1016/S0167-7012(99)00135-9 10739348

[B47] Lahlali R. Hijri M. (2010). Screening, identification and evaluation of potential biocontrol fungal endophytes against *Rhizoctonia solani* AG3 on potato plants. *FEMS Microbiol. Lett.* 311 152–159. 10.1111/j.1574-6968.2010.02084.x 20738401

[B48] Lamprecht S. Tewoldemedhin Y. (2017). Fusarium species associated with damping-off of rooibos seedlings and the potential of compost as soil amendment for disease suppression. *S. Afr. J. Bot.* 110 110–117. 10.1016/j.sajb.2016.07.009 30690970

[B49] Larran S. Perello A. Simon M. Moreno V. (2002). Isolation and analysis of endophytic microorganisms in wheat (*Triticum aestivum* L.) leaves. *World J. Microbiol. Biotechnol.* 18 683–686. 10.1023/A:1016857917950

[B50] Lee H. J. Choi G. J. Cho K. Y. (1998). Correlation of lipid peroxidation in *Botrytis cinerea* caused by dicarboximide fungicides with their fungicidal activity. *J. Agric. Food Chem.* 46 737–741. 10.1021/jf970501c 10554307

[B51] Lee S. B. Taylor J. W. (1990). “Isolation of DNA from fungal mycelia and single spores,” in *PCR protocols: A Guide to Methods and Applications*, eds Innis M. A. Gelfand D. H. Sninsky J. J. White T. J. (New York, NY: Academic Press), 282–287.

[B52] Lifshitz R. Windham M. Baker R. (1986). Mechanism of biological control of preemergence damping-off of pea by seed treatment with Trichoderma spp. *Phytopathology* 76 720–725. 10.1094/Phyto-76-720

[B53] Liu K. McInroy J. Hu C.-H. Kloepper J. W. (2017). Mixtures of plant growth-promoting Rhizobacteria enhance biological control of multiple plant diseases and plant growth promotion in the presence of pathogens. *Plant Dis.* 88 1158–1164. 10.1094/PDIS-04-17-0478-RE 30673446

[B54] Madi L. Katan T. Katan J. Henis Y. (1997). Biological control of *Sclerotium rolfsii* and *Verticillium dahliae* by *Talaromyces flavus* is mediated by different mechanisms. *Phytopathology* 87 1054–1060. 10.1094/PHYTO.1997.87.10.1054 18945040

[B55] Manhas R. K. Kaur T. (2016). Biocontrol potential of Streptomyces hydrogenans strain DH16 toward *Alternaria brassicicola* to control damping off and black leaf spot of *Raphanus sativus*. *Front. Plant Sci.* 7:1869. 10.3389/fpls.2016.01869 28018402 PMC5159428

[B56] Manzoni M. Rollini M. Bergomi S. Cavazzoni V. (1998). Production and purification of statins from *Aspergillus terreus* strains. *Biotechnol. Techniq.* 12 529–532. 10.1023/A:1008851430560

[B57] McHale A. Coughlan M. P. (1981). The cellulolytic system of *Talaromyces emersonii*. Purification and characterization of the extracellular and intracellular β-glucosidases. *Biochim. Biophys. Acta* 662 152–159. 10.1016/0005-2744(81)90236-9

[B58] Meyer J. A. Abdallah M. (1978). The fluorescent pigment of *Pseudomonas fluorescens*: biosynthesis, purification and physicochemical properties. *Microbiology* 107 319–328. 10.1099/00221287-107-2-319

[B59] Mousa W. K. Schwan A. L. Raizada M. N. (2016). Characterization of antifungal natural products isolated from endophytic fungi of finger millet (*Eleusine coracana*). *Molecules* 21:1171. 10.3390/molecules21091171 27598120 PMC6273740

[B60] Muthukumar A. Bhaskaran R. Sanjeevkumar K. (2010). Efficacy of endophytic *Pseudomonas fluorescens* (Trevisan) migula against chilli damping-off. *J. Biopestic.* 3 105–109.

[B61] Nakagawa M. Hirota A. Sakai H. Isogai A. (1982). Terrecyclic acid A, A new antibiotic from *Aspergillus terreus* I. Taxonomy, production, and chemical and biological properties. *J. Antibiot.* 35 778–782. 10.7164/antibiotics.35.778 7174531

[B62] Nakkeeran S. Kavitha K. Chandrasekar G. Renukadevi P. Fernando W. (2006). Induction of plant defence compounds by *Pseudomonas chlororaphis* PA23 and *Bacillus subtilis* BSCBE4 in controlling damping-off of hot pepper caused by *Pythium aphanidermatum*. *Biocontrol Sci. Technol.* 16 403–416. 10.1080/09583150500532196

[B63] Naureen Z. Rehman N. U. Hussain H. Hussain J. Gilani S. A. Al Housni S. K. (2017). Exploring the potentials of *Lysinibacillus sphaericus* ZA9 for plant growth promotion and biocontrol activities against phytopathogenic fungi. *Front. Microbiol.* 8:1477. 10.3389/fmicb.2017.01477 28861045 PMC5563071

[B64] Papavizas G. O’Neill N. Lewis J. (1978). Fungistatic activity of propyl-N-[adimethylaminopropyl) carbamate on Pythium spp. and its reversal by sterols. *Phytopathology* 68 1667–1671. 10.1094/Phyto-68-1667

[B65] Paulitz T. Nowak-Thompson B. Gamard P. Tsang E. Loper J. (2000). A novel antifungal furanone from *Pseudomonas aureofaciens*, a biocontrol agent of fungal plant pathogens. *J. Chem. Ecol.* 26 1515–1524. 10.1023/A:1005595927521

[B66] Peterson S. W. (2008). Phylogenetic analysis of Aspergillus species using DNA sequences from four loci. *Mycologia* 100 205–226. 10.1080/15572536.2008.11832477 18595197

[B67] Prapagdee B. Kuekulvong C. Mongkolsuk S. (2008). Antifungal potential of extracellular metabolites produced by *Streptomyces hygroscopicus* against phytopathogenic fungi. *Int. J. Biol. Sci.* 4 330–337. 10.7150/ijbs.4.330 18825279 PMC2556053

[B68] Rachid D. Ahmed B. (2005). Effect of iron and growth inhibitors on siderophores production by *Pseudomonas fluorescens*. *Afr. J. Biotechnol.* 4 697–702. 10.5897/AJB2005.000-3129

[B69] Rajendran L. Samiyappan R. (2008). Endophytic Bacillus species confer increased resistance in cotton against damping off disease caused by *Rhizoctonia solani*. *Plant Pathol. J.* 7 1–12. 10.3923/ppj.2008.1.12

[B70] Ramette A. Frapolli M. Défago G. Moënne-Loccoz Y. (2003). Phylogeny of HCN synthase-encoding hcnBC genes in biocontrol fluorescent pseudomonads and its relationship with host plant species and HCN synthesis ability. *Mol. Plant Microbe Interact.* 16 525–535. 10.1094/MPMI.2003.16.6.525 12795378

[B71] Reino J. L. Guerrero R. F. Hernández-Galán R. Collado I. G. (2008). Secondary metabolites from species of the biocontrol agent *Trichoderma*. *Phytochem. Rev.* 7 89–123. 10.1007/s11101-006-9032-2

[B72] Sadeghi A. Hessan A. Askari H. Aghighi S. Shahidi Bonjar G. (2006). Biological control potential of two Streptomyces isolates on *Rhizoctonia solani*, the causal agent of damping-off of sugar beet. *Pak. J. Biol. Sci.* 9 904–910. 10.3923/pjbs.2006.904.910

[B73] Saha B. C. Kennedy G. J. Qureshi N. Bowman M. J. (2017). Production of itaconic acid from pentose sugars by *Aspergillus terreus*. *Biotechnol. Prog.* 33 1059–1067. 10.1002/btpr.2485 28440059

[B74] Samson R. A. Visagie C. M. Houbraken J. Hong S.-B. Hubka V. Klaasen C. H. W. (2014). Phylogeny, identification and nomenclature of the genus *Aspergillus*. *Stud. Mycol.* 78 141–173. 10.1016/j.simyco.2014.07.004 25492982 PMC4260807

[B75] Schwyn B. Neilands J. (1987). Universal chemical assay for the detection and determination of siderophores. *Anal. Biochem.* 160 47–56. 10.1016/0003-2697(87)90612-9 2952030

[B76] Sharifi-Tehrani A. Omati F. (1999). “Biocontrol of *Phytophthora capsici* the causal agent of pepper damping-off by antagonistic bacteria,” in *Proceedings of the 51st International Symposium on Crop Protectionm Gent*, Belgium.

[B77] Silvestro D. Michalak I. (2012). RaxmlGUI: a graphical front-end for RAxML. *Org. Divers. Evol.* 12 335–337. 10.1007/s13127-011-0056-0

[B78] Singh S. Mathur N. (2010). Biological control of root-knot nematode, *Meloidogyne incognita* infesting tomato. *Biocontrol Sci. Technol.* 20 865–874. 10.1080/09583157.2010.487935

[B79] Sivan A. Elad Y. Chet I. (1984). Biological control effects of a new isolate of *Trichoderma harzianum* on *Pythium aphanidermatum*. *Phytopathology* 74 498–501. 10.1094/Phyto-74-498

[B80] Sreedevi K. VenkateswaraRao J. Narasu L. Md F. (2017). Strain improvement of *Aspergillus terreus* for the enhanced production of lovastatin, a HMG-COA reductase inhibitor. *J. Microbiol. Biotechnol. Res.* 1 96–100.

[B81] Szczech M. Shoda M. (2004). Biocontrol of rhizoctonia damping-off of tomato by *Bacillus subtilis* combined with *Burkholderia cepacia*. *J. Phytopathol.* 152 549–556. 10.1111/j.1439-0434.2004.00894.x

[B82] Tamura K. Stecher G. Peterson D. Filipski A. Kumar S. (2013). MEGA6: Molecular Evolutionary Genetics Analysis version 6.0. *Mol. Biol. Evol.* 30 2725–2729. 10.1093/molbev/mst197 24132122 PMC3840312

[B83] Tarafdar J. Rao A. Bala K. (1988). Production of phosphatates by fungi isolated from desert soils. *Folia Microbiol.* 33 453–457. 10.1007/BF02925770

[B84] Thieken A. Winkelmann G. (1992). Rhizoferrin: a complexone type siderophore of the mocorales and entomophthorales (Zygomycetes). *FEMS Microbiol. Lett.* 94 37–41. 10.1111/j.1574-6968.1992.tb05285.x 1387861

[B85] Thrane C. Tronsmo A. Jensen D. F. (1997). Endo-1, 3-β-glucanase and cellulase from *Trichoderma harzianum*: purification and partial characterization, induction of and biological activity against plant pathogenic Pythium spp. *Eur. J. Plant Pathol.* 103 331–344. 10.1023/A:1008679319544

[B86] Wakelin S. A. Warren R. A. Harvey P. R. Ryder M. H. (2004). Phosphate solubilization by Penicillium spp. closely associated with wheat roots. *Biol. Fertil. Soils* 40 36–43. 10.1007/s00374-004-0750-6

[B87] Wang Y. Zheng J. Liu P. Wang W. Zhu W. (2011). Three new compounds from *Aspergillus terreus* PT06-2 grown in a high salt medium. *Mar. Drugs* 9 1368–1378. 10.3390/md9081368 21892351 PMC3164379

[B88] Waqas M. Khan A. L. Hamayun M. Shahzad R. Kim Y.-H. Choi K.-S. (2015). Endophytic infection alleviates biotic stress in sunflower through regulation of defence hormones, antioxidants and functional amino acids. *Eur. J. Plant Pathol.* 141 803–824. 10.1007/s10658-014-0581-8

[B89] Weller D. M. (1988). Biological control of soilborne plant pathogens in the rhizosphere with bacteria. *Annu. Rev. Phytopathol.* 26 379–407. 10.1146/annurev.py.26.090188.002115

[B90] Weller D. M. Cook R. J. (1986). “Suppression of root diseases of wheat by fluorescent pseudomonads and mechanisms of action,” in *Iron, Siderophores, and Plant Diseases. NATO ASI Series (Series A: Life Sciences)* Vol. 117 ed. Swinburne T. R. (Boston, MA: Springer), 99–107.

[B91] Whipps J. M. (2001). Microbial interactions and biocontrol in the rhizosphere. *J. Exp. Bot.* 52 487–511. 10.1093/jxb/52.suppl_1.487 11326055

[B92] White T. J. Bruns T. Lee S. Taylor J. (1990). “Amplification and direct sequencing of fungal ribosomal RNA genes for phylogenetics,” in *PCR protocols: A Guide to Methods and Applications*, eds Innis M. A. Gelfand D. H. Sninsky J. J. White T. J. (New York, NY: Academic Press), 315–322.

[B93] Xiao J. Zhang Q. Gao Y.-Q. Tang J.-J. Zhang A.-L. Gao J.-M. (2014). Secondary metabolites from the endophytic *Botryosphaeria dothidea* of *Melia azedarach* and their antifungal, antibacterial, antioxidant, and cytotoxic activities. *J. Agric. Food Chem.* 62 3584–3590. 10.1021/jf500054f 24689437

[B94] Yedidia I. Benhamou N. Chet I. (1999). Induction of defense responses in cucumber plants (*Cucumis sativus* L.) by the biocontrol agent *Trichoderma harzianum*. *Appl. Environ. Microbiol.* 65 1061–1070. 10049864 10.1128/aem.65.3.1061-1070.1999 PMC91145

[B95] Zhang A. M. Zhao G. Y. Gao T. G. Wang W. Li J. Zhang S. F. (2013). Solubilization of insoluble potassium and phosphate by *Paenibacillus kribensis* CX-7: a soil microorganism with biological control potential. *Afr. J. Microbiol. Res.* 7 41–47. 10.5897/AJMR12.1485

