# Supplementary figures and images for: Aspergillus terreus Inhibits Growth and Induces Morphological Abnormalities in Pythium aphanidermatum and Suppresses Pythium-Induced Damping-Off of Cucumber
Source: Front Microbiol. 2018 Feb 1;9:95. doi: 10.3389/fmicb.2018.00095 (PMC5799290; doi:10.3389/fmicb.2018.00095)

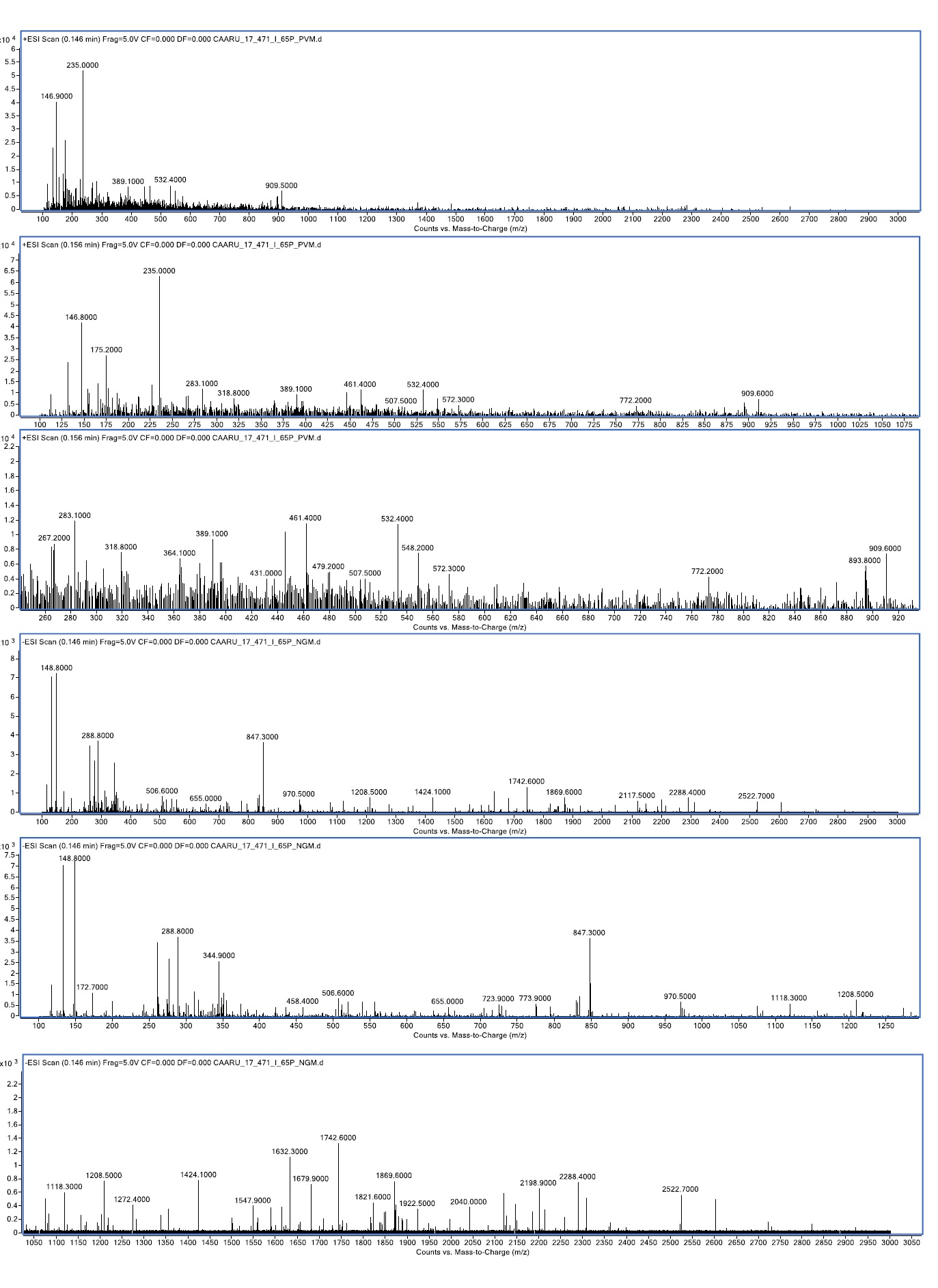

Supplement: FIGURE S1 — Mass spectrum (m/z) of Aspergillus terreus 65P isolate culture filtrate (CF). (A–C) Shows positive ESI scan (at 0.146 min and 100–3000 m/z), (at 156 min and 100:1075 m/z), (at 156 min and 260–920 m/z), respectively, while (D–F) show negative ESI scan (at 0.146 min and 100:3000 m/z), (at 0.146 min and 100:1250 m/z), (at 0.146 min and 1050:3000 m/z), respectively. [file Image_1.JPEG]

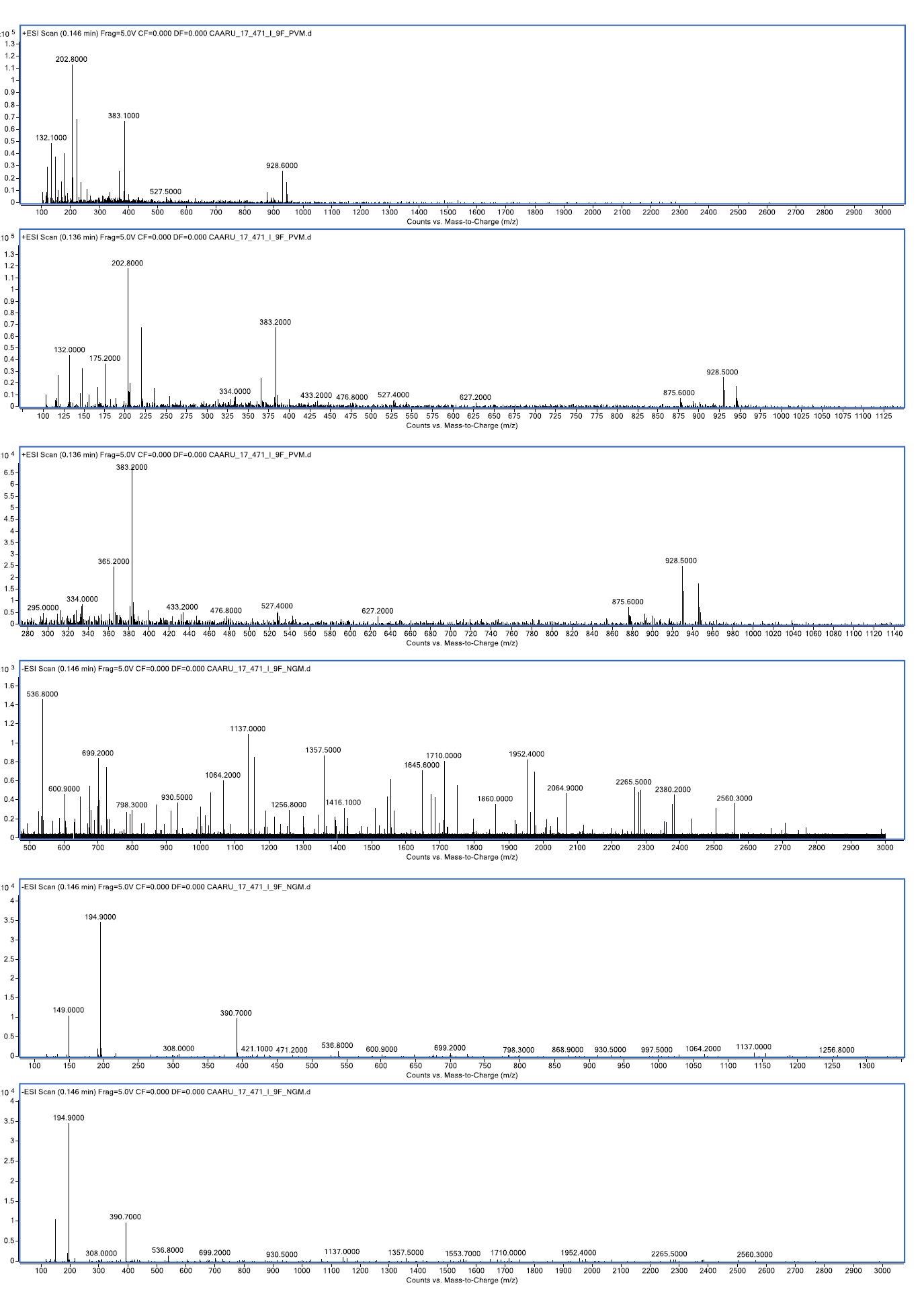

Supplement: FIGURE S2 — Mass spectrum (m/z) of A. terreus 9F isolate CF. (A–C) Shows positive ESI scan (at 0.146 min and 100–3000 m/z), (at 136 min and 100:1125 m/z), (at 136 min and 280–1140 m/z), respectively, while (D–F) show negative ESI scan (at 0.146 min and 100:3000 m/z), (at 0.146 min and 100:1300 m/z), (at 0.146 min and 500:3000 m/z), respectively. [file Image_2.JPEG]
